# Cervicovaginal Bacteriology and Antibiotic Sensitivity Patterns among Women with Premature Rupture of Membranes in Mulago Hospital, Kampala, Uganda: A Cross-Sectional Study

**DOI:** 10.1155/2017/9264571

**Published:** 2017-02-09

**Authors:** Milton W. Musaba, Mike N. Kagawa, Charles Kiggundu, Paul Kiondo, Julius Wandabwa

**Affiliations:** ^1^Mbale Regional Referral & Teaching Hospital, P.O. Box 921, Mbale, Uganda; ^2^School of Medicine, College of Health Sciences, Makerere University, P.O. Box 7072, Kampala, Uganda; ^3^Mulago National Referral & Teaching Hospital, P.O. Box 7051, Kampala, Uganda; ^4^Faculty of Health Sciences, Busitema University, P.O. Box 1460, Mbale, Uganda

## Abstract

*Background*. A 2013 Cochrane review concluded that the choice of antibiotics for prophylaxis in PROM is not clear. In Uganda, a combination of oral erythromycin and amoxicillin is the 1st line for prophylaxis against ascending infection. Our aim was to establish the current cervicovaginal bacteriology and antibiotic sensitivity patterns.* Methods*. Liquor was collected aseptically from the endocervical canal and pool in the posterior fornix of the vagina using a pipette. Aerobic cultures were performed on blood, chocolate, and MacConkey agar and incubated at 35–37°C for 24–48 hrs. Enrichment media were utilized to culture for GBS and facultative anaerobes. Isolates were identified using colonial morphology, gram staining, and biochemical analysis. Sensitivity testing was performed via Kirby-Bauer disk diffusion and dilution method. Pearson's chi-squared (*χ*^2^) test and the paired* t*-test were applied, at a *P* value of 0.05.* Results*. Thirty percent of the cultures were positive and over 90% were aerobic microorganisms. Resistance to erythromycin, ampicillin, cotrimoxazole, and ceftriaxone was 44%, 95%, 96%, and 24%, respectively. Rupture of membranes (>12 hrs), late preterm, and term PROM were associated with more positive cultures.* Conclusion*. The spectrum of bacteria associated with PROM has not changed, but resistance to erythromycin and ampicillin has increased.

## 1. Background

Prelabour rupture of membranes is the commonest antecedent to preterm and term labour [1]. The aetiology of preterm delivery has been attributed to a number of causes such as decidual hemorrhage, inflammation of the fetal membranes, activation of the maternal-fetal hypothalamic pituitary axis, structural abnormalities like cervical incompetence, pathologic distension of the uterus, and uterine abnormalities [2]. However, there is strong epidemiological and biochemical evidence which links preterm and term delivery to ascending infection of the female genital tract [3].

The incidence of maternal and neonatal sepsis ranges from 1 to 25% depending on the duration of rupture of membranes and gestation age [4] or even higher in low resource settings [5]. In Uganda, 22% of the maternal deaths are due to sepsis [6] and sepsis is the 2nd major cause of neonatal mortality [7]. Premature rupture of membranes at or near term complicates 3% of all pregnancies in Mulago Hospital [8]. Anecdotal evidence suggests that most of these patients suffer adverse obstetric outcomes due to sepsis.

Several organisms have been variably associated with PROM in different parts of the world [1]. Previous culture and sensitivity studies in this settings have involved sick neonates and patients with surgical site infections [9, 10]. Only one found that 13.3% of 180 women with PROM in Mulago Hospital had positive amniotic fluid cultures with descending order isolates of* Escherichia coli*, beta hemolytic* Streptococcus*,* Proteus* species, and* Peptostreptococcus*, although the antibiotic sensitivity patterns were not done [8].

The choice of antibiotics for prophylaxis should be based on culture and sensitivity patterns as well as epidemiological patterns, but because of limited access to reliable laboratory services in Uganda, broad spectrum antibiotics are given empirically. A combination of oral erythromycin (250 mg) and amoxicillin (500 mg) for 7 days is the 1st line for prophylaxis against ascending genital tract infection following PROM (MOH 2012). It is not clear if this recommendation is based on current culture and sensitivity patterns. In Africa, resistance to commonly used antibiotics has greatly increased over the last 20 years [9]; so we are no longer sure if this recommendation is still relevant. A recent Cochrane review which included 22 studies from high resource settings concluded that the antibiotic of choice for prophylaxis in PROM is not clear [11]. Probably the associated organisms have changed or developed resistance, hence the need to establish the current bacterial culture and sensitivity patterns in this population of patients. This information may inform our choice of antibiotics for prophylaxis against ascending infection following PROM and probably contribute to the body of evidence needed to either revise or maintain the current guideline for management of PROM.

The aim of this study was to establish the current cervicovaginal bacteriology and antibiotic sensitivity patterns.

## 2. Materials and Methods

### 2.1. Study Design

A cross-sectional study was conducted on the labour suite at Mulago National Referral and Teaching Hospital Uganda between January and May, 2013.

### 2.2. Study Setting

Mulago Hospital is the national referral hospital for Uganda and a teaching hospital for Makerere University. The hospital averages 30,000 births per year. In 2011, 868 patients with PROM were admitted in Mulago Hospital, none of them had culture and sensitivity patterns done and the pregnancy outcomes related to sepsis were not easy to ascertain because of poor documentation.

### 2.3. Sample Size Calculation

The Leslie Kish (1965) formula for determining single proportions was used. *P* was the percentage of positive amniotic fluid cultures among patients with PROM in Mulago National hospital, using 13.3% [8].

### 2.4. Sampling

We consecutively enrolled 196 women with a diagnosis of PROM and a viable foetus at or after 28 weeks of gestation, over a 5 months' period. Ethical approval was obtained from the School of Medicine Research and Ethics Committee, Makerere University College of Health Sciences, and the Uganda National Council for Science and Technology. Each participant was requested to give written informed consent. An interviewer administered questionnaire was used to collect data on patient demographics and clinical features [12].

Using an aseptic technique, 5 mLs of amniotic fluid was collected from both the endocervical canal and the pool in the posterior fornix of the vagina using a pipette and transported to the microbiology laboratory within 30 minutes for analysis. Specimen was transported in 3 separate containers: 1 mL inoculated into a selective Todd-Hewitt enrichment media to culture for GBS in a glass bottle; 1 mL inoculated into thioglycolate enrichment media to culture for facultative anaerobes in a glass bottle; 3 mL in a sterile plastic container. The aerobic culture was performed on blood, chocolate, and MacConkey agar incubated at 35–37° for 24–48 hours [12]. Colonial morphology, gram staining, and biochemical analysis were used to identify the isolates. The Kirby-Bauer disk diffusion and dilution methods were used to test for sensitivity of the isolates against nitroimidazoles, penicillins, cephalosporins, sulfonamides, aminoglycosides, quinolones/fluoroquinolones, macrolides, and tetracyclines, as specified in the manual of clinical microbiology (Jorgensen, Pfaller et al. 2015). Ten percent of the samples were taken to 2 laboratories (Medical School Laboratory and MBN diagnostic laboratories) for quality control purposes [12].

Data was entered into Epidata version 3.1 and then exported into Stata version 12 for analysis. The proportion of positive amniotic fluid cultures was computed; statistical significance was tested via the chi (*χ*^2^) test [12]. The paired* t*-test was applied to measure the significance of the factors associated with positive cultures (*P* = 0.05).

## 3. Results

Fifty-eight (30%) of the 196 cervicovaginal cultures were positive. A quarter of the participants had received an antibiotic prior to admission and the most commonly used antibiotics were ceftriaxone (29), ampicillin (13), erythromycin (9), and metronidazole (9) (Table 1).

Sixty-seven isolates were identified from the 58 positive cultures, 63% were gram positive aerobic bacteria, and only 5 (7.5%) were anaerobes (Table 2).

Resistance to most commonly used broad spectrum antibiotics (ceftriaxone, ampicillin, cotrimoxazole, and erythromycin) was notably high. It is only the less commonly used and more expensive antibiotics like vancomycin and meropenem that showed the highest levels of sensitivity to the isolated antibiotics (Table 3).

Rupture of membranes for >12 hrs and late preterm and term PROM mothers were associated with a higher percentage of positive cultures. On the other hand prior use of any antibiotic following rupture of membranes and a positive HIV status were associated with fewer positive cultures (Table 4).

## 4. Discussion

In the current study, we found that the proportion of positive cervicovaginal cultures has doubled since 1996 [8]. This could be attributed to the use of enrichment broths to increase the yield of specific bacterial isolates. A review of 18 published studies involving 1,727 women with PROM in which amniotic fluid was obtained by amniocentesis; a 32.4% rate of positive cultures was reported for women not in labour and 75% for those admitted in labour [13]. Despite the differences in setting and techniques employed, the rate of positive cultures is similar.

The spectrum of bacteria associated with PROM has not changed much over time [8] and it is similar to those isolated from neonates with sepsis in this hospital [7].

We did not isolate any GBS despite the fact that an enrichment medium was used to favour its growth. It has been strongly associated with neonatal sepsis following vertical transmission. This could be so because either we used an antiseptic before introducing a speculum into the vagina or the site, or timing and nature of specimen collected were not ideal [14]

The yield of anaerobic bacteria was very poor, even though specific measures like the use of enrichment media for transportation of cervicovaginal samples were employed in this study. Several studies from high income settings have strongly associated them with PROM [4], so it would be interesting to study the whole picture in a better resourced laboratory before drawing conclusions [12].

Resistance to commonly used antibiotics is high which is consistent with reports of increasing antibiotic resistance in resource limited setting due to irrational use of antibiotics [9]. Due to limitations in accessing laboratory services in Uganda and other similar settings, there is a recourse to empirical use of broad spectrum antibiotics for prophylaxis against ascending genital tract infections following PROM [12]. The findings of high resistance to erythromycin and ampicillin, the 2 antibiotics recommended by MOH in Uganda, raise concerns regarding their usefulness as 1st choice prophylactic antibiotics. It is possible that either the organisms have developed resistance over time or the associated organisms have changed. Moving forward, trials need to be undertaken to identify the most appropriate antibiotic for prophylaxis following PROM in Uganda.

Although this study was not powered to determine associated factors, it was noted that there were more positive cultures when the duration of rupture of membranes was longer than 24 hrs but this result was not statistically significant; *P* = 0.055. It is also important to note that as early as 12 hrs after ROM 21% of 77 cultures were positive (Table 4). In this setting, we probably need to initiate prophylaxis early even before the recommended 18 hrs for prolonged ROM. A similar trend was also noted with the gestation age; there were more positive cultures seen with decreasing gestation age. This is consistent with other studies that have identified these two factors as important risk factors for ascending genital tract infection following PROM [15]. Further analysis revealed that mothers with late preterm and term PROM were more likely to have positive cultures compared to mothers with extreme preterm PROM. Both of them were statistically significant *P* = 0.007 and *P* = 0.000, respectively (Table 5).

Prior use of antibiotics following PROM was associated with fewer positive cervicovaginal cultures, which is expected. Although in this current study, only 23.5% of the participants reported use of antibiotics prior to admission following PROM.

## 5. Conclusions

The proportion of positive cervicovaginal cultures in Mulago National Referral and Teaching Hospital has more than doubled, but the spectrum of associated bacteria isolated has not changed since 1996. Resistance to the most commonly used antibiotics in our setting is high. There is a need for clinical trials to identify the most appropriate antibiotic for prophylaxis in PROM.

## Figures and Tables

**Table 1:** Sociodemographic and clinical characteristics (*n* = 196).

Characteristic	Frequency (%)
Mean age	25 (16–47) years
Referral status	
Yes	54 (27.6)
No	142 (72.4)
Gestation age	
Extreme preterm (28–31 weeks)	19 (10)
Late preterm (32–36 weeks)	52 (27)
Term (>37 weeks)	125 (64)
Gravidity	
Primigravida	80 (40)
2-3	60 (31)
>3	56 (29)
Prior antibiotic use	
Yes	46 (23.5)
No	150 (76.5)
Duration of ROM	
<12 hrs	77 (39)
12–24 hrs	20 (10)
>24 hrs	99 (51)

**Table 2:** Isolates from the positive cervicovaginal cultures.

	Number
Gram negative organism	
*Bacteroides* species^*∗*^	3
*Citrobacterfreundii*^*∗∗*^	2
*Enterobacterspecies*	1
*Escherichiacoli*	12
*KlebsiellaPneumoniae*	7
Gram positive organisms	
*Clostridiumtetani*^*∗*^	1
Coagulase-negative *Staphylococcus*	9
Coryneform bacteria	1
*Enterococcus species*	3
*Staphylococcus aureus*	13
*Streptococcus pyogenes*	8
*Streptococcuspneumoniae*	1
*Streptococcusspecies*	1
*Viridiansstreptococcus*^*∗∗*^	3
*Listeriamonocytogenes*	1
Positive rods isolated^*∗*^	1
*Total*	*67*

^*∗*^Anaerobe. ^*∗∗*^Facultative anaerobe.

**Table 3:** Antibiotic sensitivity profile of positive cervicovaginal cultures.

Antibiotic	Sensitive (%)	Resistant (%)	Intermediate (%)	*Total*
Ceftriaxone/cefotaxime	16 (76)	5 (24)	0	*21*
Cotrimoxazole	1 (4)	26 (96)	0	*27*
Erythromycin	15 (56)	10 (37)	2 (7)	*27*
Vancomycin	20 (95)	1 (5)	0	*21*
Chloramphenicol	23 (62)	14 (38)	0	*37*
Clindamycin	6 (40)	7 (47)	2 (13)	*15*
Ampicillin	1 (5)	20 (95)	0	*21*
Penicillin G	9 (38)	15 (63)	0	*24*
Co-amoxiclav	3 (17)	15 (83)	0	*18*
Tetracycline	6 (21)	22 (79)	0	*28*
Meropenem	6 (100)	0	0	*6*
Ciprofloxacin	21 (66)	11 (34)	0	*32*
Oxacillin	1 (7)	14 (93)	0	*15*
Ofloxacin	4 (80)	1 (20)	0	*5*
Cefazolin	3 (23)	9 (69)	1 (8)	*13*
Cefuroxime	3 (19)	13 (81)	0	*16*
Gentamicin	12 (46)	12 (46)	2 (8)	*26*
Ceftazidime	2 (100)	0	0	*2*
Nitrofurantoin	1 (100)	0	0	*1*
Cefotaxime-clavulanic acid	1 (100)	0	0	*1*

**Table 4:** Factors associated with cervicovaginal culture and sensitivity patterns.

Characteristic	Positive cultures	Negative cultures	Odds ratio (CI)	*P* values
Referral status				0.488
Yes	14 (26%)	40 (74%)	0.78 (CI 0.49–1.38)	
No	44 (31%)	98 (69%)		
Mean maternal BP	119/74	116/75	Mean = 117/75	
Mean radial pulse	83	85	Mean = 85	
Mean axillary temp.	37	37	Mean = 37	
Gestation age (weeks)				
Extreme preterm (28–31)	8 (42%)	11 (58%)	1.4 (CI 1.3–1.6)	0.000
Late preterm (32–36)	16 (31%)	36 (39%)		
Term (>37)	34 (27%)	91 (73%)		
Gravidity				
Primigravida	24 (30%)	56 (70%)	1.12 (CI 0.77–1.62)	0.343
2-3	14 (23%)	46 (77%)		
>3	20 (36%)	36 (64%)		
Duration of ROM				
<12	16 (21%)	61 (79%)	1.33 (CI 0.96–1.85)	0.055
12–24	9 (45%)	11 (55%)		
>24	33 (33%)	66 (67%)		
HIV serology				
Negative	50 (29%)	123 (71%)	0.76 (CI 0.30–1.92)	0.562
Positive	8 (35%)	15 (65%)		
Prior antibiotic use				
Yes	9 (20%)	37 (80%)	0.50 (CI 0.22–1.13)	0.089
No	49 (33%)	101 (67%)		
Prior steroid use				
Yes	7 (41%)	10 (59%)	1.76 (CI 0.63–4.90)	0.274
No	51 (28%)	128 (72%)		
Regular medication use (ART)				
Yes	6 (35%)	11 (65%)	1.33 (CI 0.47–3.80)	0.59
No	52 (29%)	127 (71%)		
Mean FHR				
<120	0 (0%)	2 (100%)	1.30 (CI 0.37–4.54)	0.654
120–160	55 (30%)	129 (70%)		
>160	3 (30%)	7 (70%)		
Gram stain result				
Positive	38 (50%)	38 (50%)	0.2 (CI 0.10–0.40)	<0.001
Negative	20 (17%)	100 (83%)		

**Table 5:** Gestation age and cervicovaginal cultures of mothers with PROM.

	Odds ratio	*P* value	95% CI	
Extreme preterm (28–31) R	1.0			
Late preterm (32–36) versus extreme preterm (28–31)	2.3	0.007	1.2	4.1
Term (>37) versus extreme preterm (28–31)	2.7	0.000	1.8	4.0

## Abbreviations

PROM: Premature rupture of membranes
C & S: Culture and sensitivity patterns
GBS: Group B* Streptococcus*
MOH: Ministry of Health.
